# Outpatient rehabilitation resources and medical expenditure in children with attention-deficit hyperactivity disorder in Taiwan

**DOI:** 10.1371/journal.pone.0199877

**Published:** 2018-06-28

**Authors:** Hsing-Jung Li, Chao-Chan Kuo, Yi-Chien Yao, Ching-Hong Tsai, Philip C. Chow, Ying-Chun Li

**Affiliations:** 1 Department of Child & Adolescent Psychiatry, Kai-Syuan Psychiatric Hospital, Kaohsiung City, Taiwan; 2 Institute of Health Care Management, Department of Business Management, National Sun Yat-Sen University, Kaohsiung City, Taiwan; 3 Department of Adult Psychiatry, Kai-Syuan Psychiatric Hospital, Kaohsiung City, Taiwan; 4 Department of Psychiatry, Conde de São Januário Central Hospital, Macau, China; Harvard Medical School, UNITED STATES

## Abstract

Attention-deficit hyperactivity disorder (ADHD) is a neurodevelopmental disorder in children. This study investigated the use of rehabilitation treatment in Taiwan. We selected children aged 3–12 years from the National Health Insurance Research Database from 2008 to 2012 and included them in the analysis. The children who received a diagnosis according to the International Classification of Diseases, Ninth Revision, Clinical Modification were divided into two groups: ADHD and non-ADHD. We used the chi-squared test, independent sample *t* test, and multiple regression analysis to conduct the analysis. The utilisation of rehabilitation resources was higher in the ADHD group than in the non-ADHD group. The number of school-aged children with ADHD was higher than the number of preschool-aged children (*p* < 0.001). The highest utilisation of rehabilitation resources was observed in clinics (*p* < 0.001). In terms of region, Taipei exhibited the highest utilisation of rehabilitation resources, and the East exhibited the lowest resource utilisation (*p* < 0.001). Prediction of the use of rehabilitation resources, average cost, average frequency of visits, and total annual cost was affected by factors such as the average frequency of rehabilitation use, demographic characteristics, and the hospital characteristics and location (*p* < 0.001). The number of children with ADHD and rehabilitation use are increasing yearly; however, limitations in payment restrict the growth of rehabilitation resource use in hospitals. Supplementation of rehabilitation resources at clinics accounts for more than 60%, however, the total annual cost is less than what is observed for hospitals (*p* < 0.001). Policies should be established to aid in the early detection and treatment of children with ADHD to improve treatment outcomes and reduce the family burden and treatment expenditure in the future.

## Introduction

Attention-deficit hyperactivity disorder (ADHD) is one of the most common mental and neurodevelopmental disorders in children. Children with ADHD exhibit numerous associated symptoms and impairments in development, socialisation, emotional and cognitive functioning, as well as behaviour problems [1]. ADHD has three core symptoms: diminished ability to sustain attention, increased impulsivity, and hyperactivity. ADHD symptoms typically present by the age of 12 years. To confirm the ADHD diagnosis, these symptoms must present in at least two settings such as school and home and must interfere with the development of appropriate social and academic functions [2]. The prevalence rate of ADHD is 5%–8% in school children, with a continuing diagnosis rate of 60%–85% into adolescence and up to 60% into adulthood. The prevalence rate in adults is 3%–5% [3,4]. ADHD has been associated with an elevated prevalence of comorbid diagnoses and problems such as substance abuse, oppositional defiant disorder and depression and anxiety disorders, social difficulty, marital discord, learning disabilities, criminality, accidental injury, and speech problems [5,6,7,8,9]. The most effective treatment for ADHD by clinical evidence is use of medications; however, behavioural treatment or counselling to learn coping skills and adaptive behaviours, parental education, and remedial education and mental support are also essential [10].

Preschoolers with ADHD incurred 17.6 times higher average costs per annum than preschoolers without ADHD. A study reported an average cost of £562 for those with ADHD compared with £30 for controls [11]. Preschool children with ADHD were more likely to receive individual and multiple rehabilitation services. Higher rates of service utilisation translated into increased costs for individual speech and occupational therapy services and special education, with the exception of physical therapy [12]. Children with ADHD used more medical resources, and most of the expenses were for non-ADHD-related treatments [13].

Page et al. [14] suggested that treatment initiation with behaviour modification rather than medication is a more cost-effective option for treating children with ADHD. In the United States, the annual costs involved in ADHD are substantial and have a large economic impact [15]. In 2005, the cost of ADHD in children and adolescents was $14,576 per individual. The annual societal cost of ADHD in childhood and adolescence is $42.5 billion [16]. Doshi et al. [17] estimated that national annual incremental costs of ADHD ranged from $143 to $266 billion. More of these costs were incurred by adults ($105–$194 billion) than by children and adolescents ($38–$72 billion). The largest cost categories in adults were productivity and income losses ($87–$138 billion). The largest cost categories in children were health care ($21–$44 billion) and education ($15–$25 billion) [17].

In 2002, the first study on the use of health care costs of ADHD in Germany was conducted, reporting an average annual cost of €142 million. These values are considerably lower than the values calculated in the United States [18]. Another German study indicated that the average age of diagnosis is 12.9 years, and the cost of the first year after diagnosis is greater than that of the previous year. Although multimodal treatment is the most effective treatment method, the proportion of people that use this treatment type remains low (10%) because the time and money invested in the treatment are not worth the benefits [19]. Based on a systematic literature review from Europe from 1990 to 2013 the average total ADHD-related costs ranged from €9,860 to €14,483 per patient, and annual national costs ranged from €1041 to €1529 million. The largest cost category was education (€648 million), followed by health care costs that ranged from €84 million (8%) to €377 million (25%) and social services costs of €4.3 million (0.3%–0.4%). Productivity losses of family members were €143–€339 million (14%–22%) [20]. These costs place a great economic burden on families.

There is very limited information on nonpharmacological and multimodal management for adjuvant therapy and medical costs among patients with ADHD in Taiwan. Only one cross-sectional study according to the Taiwan National Health Insurance (NHI) claims database was conducted among children with ADHD aged 0–7 years in outpatient rehabilitation care in 2009. The results showed that children with ADHD aged 6–7 years tended to incur less medical costs including rehabilitation fees and comorbidity medical fees than those aged 0–2 years [21]. Due to the limited information available the study has the following aims: 1) to examine rehabilitation care use and medical expenditures of children with ADHD aged 3–12 years by using data collected from NHI beneficiaries from 2008 to 2012 in Taiwan; and 2) investigate factors associated with these patterns.

## Methods

Data from the NHI claims database from 2008 to 2012 were analysed. These data included sex, date of birth, health insurance identity, medical care setting, location, average outpatient care expenditure, number of outpatient rehabilitation visits (number of patient times outpatient frequency), annual outpatient care cost (the fee of annual outpatient claim), and discharge diagnosis of disease according to the International Classification of Diseases, Ninth Revision, Clinical Modification (ICD-9-CM) coding system. Symptoms of ADHD usually occur around the age of 3. The general diagnosis age is in kindergarten or elementary school [22]. Adolescents older than 12 years with ADHD prefer medication due to class time and heavy homework [23]. Therefore, the analysis in this study was restricted to children aged 3–12 years with at least one outpatient rehabilitation claim. The present analysis was restricted to children aged 3–12 years with at least one outpatient rehabilitation claim. The ICD-9-CM diagnostic codes of attention-deficit disorder (ADD)/ADHD were as follows: 314.0x, ADD; 314.00, ADD without hyperactivity; and 314.01, ADD with hyperactivity. The cases of ADD/ADHD were identified and diagnosed by qualified medical doctors and coded for medical billing. The children who were diagnosed using ICD-9-CM codes were divided into two groups: ADD/ADHD and non-ADHD. Children who use rehabilitation resources in the non-ADHD group included those with psychosis (290–299), neurotic disorders, personality disorders, mental retardation (317–319) and other nonpsychotic mental disorders (300–316). We evaluated the differences between these two groups in the use of rehabilitation resources and determined the relevant affecting factors. Since the National Health Insurance Research Database consists of anonymous public data released for research, this study conformed to the ethical standards established by the 2004 Declaration of Helsinki and was approved by the Institutional Review Board (IRB) of Antai Tian-Sheng Memorial Hospital (17-087-B) in Taiwan.

Data were analysed using STATA 12.0 (Stata Corp, College Station, TX, USA). The chi-squared test (χ^2^) was used to compare demographic and medical care setting characteristics and rehabilitation care expenditures in the ADHD and non-ADHD groups. The *t* test was used for evaluating outpatient care expenditure, number of outpatient rehabilitation visits, and annual outpatient care cost in children with ADHD. A generalized linear model was used to evaluate the associated factors of rehabilitation care expenditure and a negative binomial regression was conducted to estimate the number of visits in children with ADHD.

## Results

Table 1 presents the demographic characteristics of children aged 3–12 years receiving rehabilitation care. They were mostly male (*p* < 0.001), with a male: female ratio of approximately 3:1. The average age of rehabilitation resource use was 6.38 years. In the ADHD group, the proportion of school-age children was 1.9 times higher than that of the preschool-age children (*p* < 0.001) by using chi-squared test. The distribution of rehabilitation use in the ADHD group was higher than in the non-ADHD group (*p* < 0.001). Eighteen percent and 1.6% of children with ADHD had a disability card and catastrophic illness card, respectively. All of the differences were significantly different (*p* < 0.001).

**Table 1:** Demographic characteristics of 3–12-year-old children receiving rehabilitation care.

	ADHD					Non-ADHD				
Year	2008 n = 811	2009 n = 874	2010 n = 899	2011 n = 922	2012 n = 1024	2008 n = 4017	2009 n = 4059	2010 n = 4210	2011 n = 4117	2012 n = 3782
Age (%)*										
Preschool	4.18 (34.16)	4.07 (35.35)	4.08 (33.93)	4.14 (34.68)	4.23 (33.98)	3.06 (46.23)	3.08 (46.91)	3.08 (47.77)	3.39 (46.49)	3.66 (42.12)
School	7.80 (65.84)	7.93 (64.65)	7.91 (66.07)	7.98 (65.32)	7.81 (66.02)	8.96 (53.77)	8.92 (53.09)	8.94 (52.23)	8.89 (53.51)	9.02 (58.69)
Gender (%)*										
Boys	75.7	76.2	77.8	76.2	75.9	61.5	59.8	59.6	60.2	62.2
Girls	24.3	23.8	22.2	23.8	24.1	38.5	40.2	40.4	39.8	37.8
Male/Female Ratio	3.1	3.2	3.5	3.2	3.2	1.6	1.5	1.5	1.5	1.6
Health Insurance Identity (%)*										
Catastrophic illness card	1.85	1.14	1.33	1.92	1.66	4.21	3.94	3.47	3.23	3.54
Disability card	18.7	19.3	18.4	17.0	17.4	11.7	12.0	12.5	12.7	13.6
Others	79.4	79.5	80.3	81.1	81.0	84.1	84.1	84.0	84.0	82.9

*p < 0.001(comparing the overall mean for ADHD for the five years with the non-ADHD overall mean for the five years)

Table 2 shows data on children aged 3–12 years who used outpatient rehabilitation care under the NHI programme during 2008–2012. In the ADHD group, the number of children using rehabilitation treatment exhibited a yearly increase. The mean number of visits for annual rehabilitation care for ADHD was 12.3(±14.5). Among the cases used in the study, the average charge per visit of ADHD was 1846 points (1 point equalling approximately 1 New Taiwan Dollar) per person every time. The total average annual cost was approximately 18,000 points per person. Medical care expenditure was higher in the ADHD group than in the non-ADHD group (*p* < 0.001).

**Table 2:** Use of rehabilitation resources by 3–12-year-old children from the National Health Insurance Research Database.

	ADHD (n = 4530)					Non-ADHD(n = 20185)				
Year	2008	2009	2010	2011	2012	2008	2009	2010	2011	2012
Number of patient (%)	811 (16.8)	874 (17.72)	899 (17.60)	922 (19.42)	1024 (21.31)	4017 (83.2)	4059 (82.28)	4210 (82.40)	4117 (80.58)	3782 (78.69)
Average of frequency*	11.33	11.98	12.27	12.36	13.47	6.41	6.61	6.79	7.24	7.48
Average cost/claim point*/**	1626.04	1760.91	1888.82	1996.44	2012.67	1224.74	1251.86	1304.47	1470.58	1420.86
Average total annual cost/claim point*/**	15519.69	17075.32	17856.46	18677.13	20796.13	9580.44	9701.02	10073.63	11885.93	11978.58

Average of frequency: total amount of annual outpatient rehabilitation visits /annual number of patients

Average cost/claim point: the average of universal outpatient claim

Average total annual cost/claim point: Average cost times Average of frequency per patient

*p < 0.001 (comparing the overall mean for ADHD for the five years with the non-ADHD overall mean for the five years)

**1 claim point equals approximately 1 New Taiwan Dollar

Table 3 presents the characteristics of the rehabilitation resource setting. Patients were most likely to use rehabilitation resources at clinics (60%–70%; *p* < 0.001). Most of these settings were non-teaching hospitals (71%–77%; *p* < 0.001), and these cases were mostly located in northern Taiwan (Taipei and North; *p* < 0.001).

**Table 3:** Characteristics of rehabilitation resource setting.

	ADHD					Non-ADHD				
Year	2008	2009	2010	2011	2012	2008	2009	2010	2011	2012
Medical setting(%)*										
Medical centre	12.33	10.87	8.90	9.88	9.77	10.38	10.17	9.98	8.65	8.20
Metropolitan hospital	16.52	16.48	17.80	15.83	15.33	11.45	12.17	11.95	11.15	11.05
Local Hospital	9.49	13.84	12.79	11.59	10.74	7.69	8.28	7.91	7.63	8.38
Clinic	61.65	58.81	60.51	62.70	64.16	70.48	69.38	70.17	72.58	72.37
Teaching hospital(%)*										
Yes	30.09	29.29	28.25	27.92	26.76	23.15	23.90	23.21	21.23	20.81
No	69.91	70.71	71.75	72.08	73.24	76.85	76.10	76.79	78.77	79.19
Location(%)*										
Taipei	47.10	47.94	49.72	48.39	50.00	30.69	32.20	32.40	32.62	32.55
North	17.76	20.14	19.35	20.46	18.55	20.99	20.25	20.74	20.79	21.87
Central	13.93	13.50	12.01	12.80	11.43	22.08	22.15	21.76	20.50	21.60
South	10.97	9.38	9.45	8.27	9.96	9.91	10.30	9.98	11.90	9.78
Kaohsiung and Pingtung	8.51	8.01	8.23	8.77	8.98	14.09	13.13	12.35	11.97	11.92
East	1.73	1.03	1.22	1.31	1.07	2.24	1.97	2.78	2.21	2.27

*p < 0.001(comparing the overall mean for ADHD for the five years with the non-ADHD overall mean for the five years)

Table 4 presents the results of the generalized linear model analyses of outpatient rehabilitation expenditure (average cost and average total annual cost which is average cost times outpatient rehabilitation visits) and the negative binomial regression of average frequency of visits. Factors associated with rehabilitation use expenditure and visits included discharge diagnosis of ADHD, the year of rehabilitation resource use, demographic characteristics, and the hospital characteristics and location (*p* < 0.001).

**Table 4:** Results of multiple linear regression model of rehabilitation use expenditure.

	Average of frequency	Average cost	Average total annual cost
Variable(reference)	IRRs(95%CI)	β (SE)	β (SE)
Constant	7.00(6.28–7.81)**	1311.73(69.98)**	10765.35(1253.47)**
ADHD(no)	1.99(1.92–2.06)**	482.26(21.43)**	6334.49(383.92)**
**Year(2008)**			
2009	1.02(0.98–1.07)	30.35(25.80)	3.31(462.13)
2010	1.07(1.03–1.12)**	102.39(25.58)**	610.51(458.24)
2011	1.16(1.11–1.21)**	276.02(25.60)**	2524.12(458.43)**
2012	1.21(1.16–1.26)**	247.96(26.00)**	2972.97(465.70)**
**Age(preschool age)**			
School age	0.64(0.63–0.66)**	-472.86(16.56)**	-7308.91(296.62)**
**Gender (girl)**			
Boy	1.15(1.12–1.18)**	81.56(16.97)**	1054.29(303.97)**
**Identity(other)**			
Catastrophic illness card	3.39(3.16–3.64)**	1030.93(46.20)**	27405.63(827.52)**
Disability card	.11(2.99–3.23)**	830.98(24.66)**	23105.21(441.63)**
**Medical setting(Medical Centre)**			
Metropolitan hospital	1.26(1.19–1.33)**	246.22(35.05)**	6338.95(627.80)**
Local hospital	1.47(1.34–1.62)**	521.75(60.86)**	16424.44(1090.14)**
Clinic	0.61(0.55–0.68)**	-206.28(66.63)**	-5591.16(1193.42)**
**Teaching hospital(no)**			
Yes	0.84(0.77–0.93)**	164.60(60.08)**	-4160.59(1076.11)**
**Location(Taipei)**			
North	0.92(0.89–0.95)**	-49.89(22.82)*	2327.47(408.72)**
Central	0.96(0.93–1.00)*	42.17(23.05)	2531.24(412.92)**
South	1.02(0.97–1.07)	27.50(29.01)	535.83(519.58)
Kaohsiung and Pingtung	0.93(0.89–0.98)**	-21.22(27.44)	172.28(491.52)
East	0.54(0.49–0.60)**	-142.78(57.60)**	-7362.45(1031.58)**

Average of frequency: total amount of annual outpatient rehabilitation visits /annual number of patients

Average cost/claim point: the average of universal outpatient claim

Average total annual cost/claim point: Average cost times Average of frequency

Negative Binomial Regression for the Average of frequency; Generalized linear model for the Average cost and Average total annual cost.

*p < 0.05

**p < 0.01

## Discussion

ADHD has the potential to be a lifelong problem, particularly if untreated, and the burden of social costs and excessive care expenditure is significant [24]. However, in many countries, ADHD has not been diagnosed or has remained untreated, resulting in an increase in ineffective treatment and the costs incurred by this disease [25]. In the United States, the overall average annual care cost is $2.1 B [26].

In this study, the number of diagnoses from 2008 to 2011, irrespective of the number of people who used rehabilitation resources or were diagnosed as having ADHD, exhibited a yearly increase. Although the number of people who used rehabilitation resources in 2012 was the lowest in the past 5 years, the highest number of people diagnosed as having ADHD and the highest average cost of rehabilitation resource use were observed in 2012.

The male: female ratio was 3:1, similar to that in other studies [24]. Irrespective of the total annual cost, average cost, and average number of visits, boys used the rehabilitation resources more than girls (*p* < 0.05). Therefore, male sex can be used as a predictor for the subsequent use of rehabilitation resources. The exact cause of this difference remains unclear. Boys may be predominantly more hyperactive and may exhibit more externalizing symptoms, thereby resulting in a higher referral rate.

In this study, an annual total cost of 17,985 points (1 point equalling approximately 1 New Taiwan Dollar) per person was observed, exceeding the average annual outpatient expenditure of 12,000 points per person obtained from Taiwan NHI from 2003 to 2006 [27]. However, a similar result was obtained in a study in the United States in 2003, which reported an average cost of $649 per person per year and an average cost of $495 per child receiving general medical services[26].

In this study, the average age of ADHD diagnosis was 6.38 years old, and the average ages of preschool and school children were 4.14 and 7.88 years, respectively, which is consistent with the average school age of American children, reported as 7.3 years [28]. In this study, with a distinction of 6 years between the preschool and school ages, the proportion of children with ADHD using rehabilitation resources was higher in the school-age group than in the preschool-age group (*p* < 0.05). However, the overall cost of rehabilitation for the total annual cost, average cost, average number of visits, was lower in the school-age group than in the preschool-age group. This may be because students in the lower grades of elementary school have a half-day of school and thus have more time to participate in rehabilitation training programmes. The majority of the parents of children with the first diagnosis of ADHD at school age were willing to accept nondrug treatment options to ameliorate their children’s symptoms. Although the proportion of rehabilitation resource use after school age was higher, the children’s heavy workload, full school days, and lack of persistence may have been what led parents to prefer the use of medicines to nonpharmacological treatment, potentially resulting in the decline in the overall rehabilitation costs and the number of visits in school-age group. Moreover, because rehabilitation treatment fees are higher for preschool-aged children, the use of rehabilitation resources and the total annual cost, average cost, and average number of visits were higher in the preschool-age group.

In the linear regression, the total annual cost, average cost, and average number of visits were significantly related to the children having a catastrophic illness card and disability card (*p* < 0.05). Children with the catastrophic illness card can be exempted from medical costs, and those with the disability card can apply for more service supplements (such as reimbursement for transportation fees). The catastrophic illness card and disability card had more physical conditions, potentially leading to more frequent rehabilitation therapy and medical treatment and thereby increasing the cost, the number of visits, and consumption of medical resources.

In terms of hospital characteristics, most rehabilitation resources were provided at primary clinics, accounting for more than 60%, followed by metropolitan hospitals, medical centres, and local community hospitals. The demand for rehabilitation resources was presumed to be increasing in clinics, which accounted for more than half of the rehabilitation resource settings because of the increase in hospital demand. Moreover, non-teaching hospitals provided more rehabilitation resources than teaching hospitals (*p* < 0.05), which can be explained by the higher supplementation of resources by clinics.

The average cost per year, frequency of visits, and annual total cost were higher in the ADHD group than in the non-ADHD group (*p* < 0.001). The prediction of rehabilitation resource use for ADHD, the average cost, the average frequency, and the total annual cost were affected by factors such as diagnosis, the year of rehabilitation use, demographic characteristics, and hospital characteristics and location (*p* < 0.001).

In terms of the average number of visits, metropolitan hospitals and local community hospitals had more rehabilitation needs and therefore higher numbers of rehabilitation visits than medical centres. In terms of health care payments, medical centres and metropolitan hospitals received the most and clinics the least. In terms of payment for rehabilitation resources, local community hospitals had the highest average cost, total annual cost, and average number of visits, followed by primary clinics and medical centres.

Community hospitals are assumed to provide more intensive projects and follow-up treatments than the metropolitan hospitals and medical centres. Rehabilitation therapies that may have been paid for completely by a patient’s parents were not recorded in the health insurance database; therefore, the cost and frequency of visits may have been underestimated. Because of the limited resources provided at medical centres, possibly due to the total payment limitation, rehabilitation resource provision has become saturated. Children are required to wait in line to receive treatment, which limits the growth of rehabilitation resource use. Therefore, people tend to visit metropolitan or community hospitals for treatment. Due to the difference in the cost of payment from NHI and the resulting out-of-pocket expenses, although treatment supplementation is most common in clinics, because of lack of continuance, the average and total costs are the lowest at clinics.

In addition, resource supplementation was the highest in Taipei City, followed by the North, Central, South, Kaohsiung–Pingtung area, and finally the East. This result is consistent with other domestic studies [23]. Irrespective of the average cost, annual cost, and average number of visits, compared with Taipei City, the East exhibited a significant reduction in the use of medical resources. The East lacks an adequate number of providers. Although Taipei City exhibited the highest use of rehabilitation resources, the average cost, annual cost, and average number of visits there were lower than those of other cities and regions. This may be because children received treatment in the early periods with relatively mild symptoms. In addition, parents in Taipei City can select non-health insurance therapy or treatment, with out-of-pocket payment in return for a wider selection of resources. Therefore, children in Taipei had more treatment options with more favourable outcomes, follow-up rehabilitation therapy was less frequent.

The present study had some limitations. First, it was based on data from the NHI database and was subject to the following restrictions. Information was limited to rehabilitation outpatient treatment and did not include inpatient treatment details. In addition, a lack of clinical judgement was observed in the health care database because the accuracy of assessment depended entirely on the diagnosis and coding. The majority (80%) of ADHD rehabilitation therapy referrals were obtained from rehabilitation physicians and only 7% from child psychiatrists [26]; therefore, misdiagnosis or insufficient evidence for a correct diagnosis would result in inaccurate analysis and incorrect results.

Second, one declaration of outpatient rehabilitation service containing six different dates of treatment cannot represent the intensity and persistence of treatment. Some hospitals provide the therapy at the patients’ own expense, which could not be considered in this study.

Third, the information used in this study represents a secondary data analysis of the health care database maintained by the National Institutes of Health. The database lacks information on the social status of parents, family income, educational background, quality of family interaction, willingness to seek treatment, or restrictions on their own expenses.

## Conclusion

Studies on the use of nonpharmacological treatments as rehabilitation resources in Taiwan are limited. Therefore, further investigation on this topic is warranted. Currently, the number of people who use rehabilitation resources is increasing, however due to the total payment limitation, rehabilitation resource provision has become saturated which limits the growth of rehabilitation resource use in hospitals. Rehabilitation treatment requires more professional staff and more time. Due to the total payments for hospitals, it causes a reduction in the supply of rehabilitation and human resources. The value of manpower is not equal to the general payment provided by the NHI. A seriously uneven distribution of medical resources is observed in Taiwan, with the least medical resources being available in the East and the most being available in the North. Access to medical resources in remote areas remains a serious problem. The payment for clinics is less than half of that for medical centres and regional hospitals. Though the most supplement of rehabilitation resources is provided by clinics (60%) but the average annual cost is less than hospitals. This disparity reduces willingness to provide health care service supplementation from clinics. The inadequacy of rehabilitation resources critically restricts the treatment opportunities for children.

ADHD involves not only medical costs but also educational and social costs. Sufficient medical resources must be available to families to aid parents in caring for their children with ADHD and address medical and educational problems. To invest more resources in social costs, the government must develop an effective advanced policy to improve the current situation by providing sufficient assistance to children and their families to reduce future burdens on families, education, and society.
